# A novel mutation in *CYP17A1* gene leads to congenital adrenal hyperplasia: A case report

**DOI:** 10.18502/ijrm.v17i6.4817

**Published:** 2019-07-29

**Authors:** Majid Nazari, Mohammad Yahya Vahidi Mehrjardi, Nosrat Neghab, Mahdi Aghabagheri, Nasrin Ghasemi

**Affiliations:** ^1^Department of Medical Genetics, Shahid Sadoughi University of Medical Sciences, Yazd, Iran.; ^2^Medical Genetics Research Center, Shahid Sadoughi University of Medical Sciences, Yazd, Iran.; ^3^Yazd Reproductive Sciences Institute, Shahid Sadoughi University of Medical Sciences, Yazd, Iran.; ^4^Meybod Nursing School, Yazd, Iran.; ^5^Abortion Research Centre, Yazd Reproductive Sciences Institute, Shahid Sadoughi University of Medical Sciences, Yazd, Iran.

**Keywords:** Congenital adrenal hyperplasia (CAH), CYP17A1 gene, Ambiguous genitalia.

## Abstract

**Background:**

Congenital adrenal hyperplasia is a rare autosomal recessive disorder where the mutation in P450 family 17 subfamily A member 1 gene (*CYP17A1*) is involved in its etiology. The disorder represents itself with low blood levels of estrogens, androgens, and cortisol that generally couples with hypertension, Hypokalemia, sexual primary amenorrhea, infantilism and in affected individuals.

**Case:**

In this study, the *CYP17A1* gene in a 14-year-old female was examined. The karyotype of the patient was 46, XX, and the analysis of the *CYP17A1* gene by Sanger sequencing revealed a novel homozygous deletion c.1052-1054CCT which led to isolated 17,20-lyase deficiency.

**Conclusion:**

In conclusion, this study report an in-frame deletion which results in isolated 17, 20-lyase deficiency, and the mutation might be used for diagnosis in other patients with distinctive clinical symptoms

## 1. Introduction

Congenital adrenal hyperplasia (CAH) is a group of rare disorders demonstrated by a failure in one of the five enzymes responsible for cortisol production in autosomal recessive pattern (1). In adrenal glands, Pregnenolone is made from cholesterol which later can be processed to either mineralocorticoids or glucocorticoids or to sex steroids in adrenals and gonads (2).

The p450c17 enzyme is encoded by the *CYP17A1* gene located on 10q24.3 (3) and spans 6.6 kb, which comprises eight exons (4). "This gene transcribes 2.1-kb mRNA molecule, which expresses in both the adrenals and gonads and generates a 57-kDa microsomal cytochrome P450c17 enzyme. The *CYP17A1* enzyme catalyzes both steroid 17-hydroxylase and 17,20-lyase activities" (5). Enzymatic failure of the P450c17 enzyme leads to both glucocorticoids and sex steroids deficiencies. After a reduction in blood glucocorticoids levels due to P450c17 defect, anterior pituitary tries to compensate for the insufficiency of glucocorticoids levels by producing extra Adrenocorticotropic hormone (ACTH). This results in the extra generation of steroid precursors and elevated ACTH level, which leads to some *Congenital* adrenal hyperplasia and result in hypertension, hypokalemia, and a suppressed renin-angiotensin system (6, 7). Moreover, due to the impairments in *CYP17A1*, the mineralocorticoid precursors (corticosterone and 11-deoxycorticosterone) accumulate, which demonstrate glucocorticoid activity, therefore defect in P450c17 does not associate with adrenal crisis, rather than other CAH variants (8). Mutations in the *CYP17A1* gene are the rarest defects in CAH that yields to steroid 17-hydroxylase and 17,20-lyase deficiencies (9). Several mutations in the CYP17 gene have been reported that cause either complete or combined17-hydroxylase/17,20-lyase or isolated 17, 20-lyase enzyme deficiencies (10-14).

The purpose of this study was to investigate the molecular defects in *CYP17A1* gene and its relationship with Congenital adrenal hyperplasia.

## 2. Case presentation

A 14-year-old female, the first child of consanguineous parents with normal family history was referred to the genetic clinic with high blood pressure, ambiguous genitalia, and lack of pubertal development. The blood sample was taken after receiving written informed consent from her parents. No pubic or axillary hair was seen by physical examination, and she had no clinical symptoms of Turner syndrome with 46, XX karyotype. In the sonographic survey, uterus was infantile. She was hypertensive (150/90 mmHg, 50th percentile for age) with high gonadotropins levels (LH, 19 mU/mL; FSH, 34 mU/mL). Moreover, low peripheral concentrations of sex steroids were seen (Table I).

### Sequencing of *CYP17A1* gene

Genomic DNA was purified from peripheral blood leukocytes (PBL) using QIAGEN Mini Blood kit. All the exons of *CYP17A1* genewere proliferated by PCR (primers listed in Table II), which were designed with Primer3 software (http://primer3.sourceforge.net). All the PCR products were sequenced in both directions by sanger sequencing.

### Mutational analysis 

**Figure 1:**
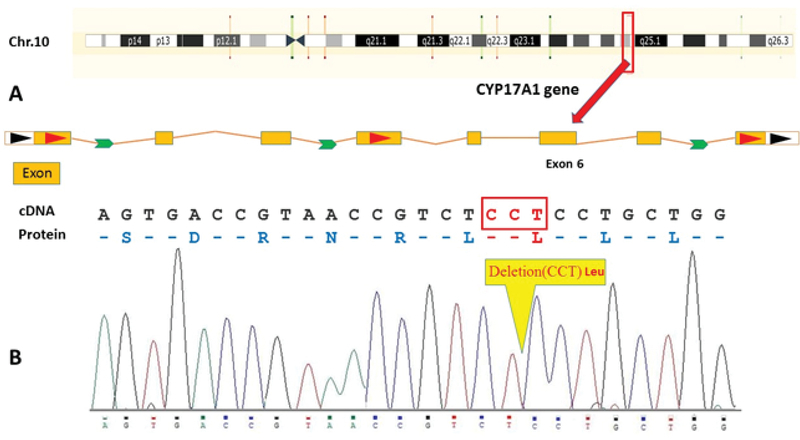
(A) Schematic representation of *CYP17A1* gene location on chr.10. (B) Sanger sequence chromatogram of the *CYP17A1* gene show three nucleotide deletion position 1552–1554 within exon 6 (deletion of 351Leu in protein and CCT deletion on cDNA sequence).

As shown in Figure 1, a new in-frame homozygous deletion c.1052-1054CCT in exon 6 was identified that reported for the first time. 17α-hydroxylase deficiency was first pronounced by Biglieri and colleagues (15), who was phenotypically female and presented with sexual infantilism, primary amenorrhea and hypertension.

**Table 1:** Clinical and hormonal characteristics


Blood pressure (mmHg)	150/90
Karyotype	46,XX
Tanner stage (breast/pubic hair)	B1P1
ACTH (0-60 pg/mL)	76
Cortisol (5-25 μg/dL)	17
LH (Female, 12-18 yr: 0.1-10 mU/mL)	19
FSH (Female, 12-18 yr: 0.3-9 mU/mL)	34
Estradiol (30-120 pg/mL)	15
DHEA (350-4300 ng/mL)	52
Progesterone (0.1-1.3 ng/mL)	4.6

**Table 2:** Primers for *CYP17A1* amplification


**Exon**	**Primers**	**Sequences (5'-3')**	**Areas**	**Product size (bp)**
1	1F	CACTGCTGTCTATCTTGCC	1802-2277	476
	1R	CCTTCACATCATCCCACTA	
2	2F	AGGGACCAGAGGTGTAAG	3730-4070	341
	2R	GCAGCAGTAGCCAAGAA	
3	3F	AGGGTGCTGATTCATTTC	4132-4544	413
	3R	GCAGAGGAGGTAGAGGTG	
4	4F	CGCTTGATGTTTGATTGA	4819-5214	396
	4R	CACCCTGCTCTTGTGATT	
5	5F	ACAGAAGTATGGCAGGAGT	5776-6289	514
	5R	CCAGAGTAGGTTGGAGGT	
6	6F	ACTGGGAAGGGACTGGA	6182-6496	315
	6R	GGCTAGATGTCACTGGGAG	
7	7F	AGTGGGAATGAGGGAGTA	7244-7599	356
	7R	GTCAACAGGTCGGTATAGTT	
8	8F	TCAACCAGGGCAGAACC	7914-8359	446
	8R	GGAAGAATGGCGGAGAA	
F: Forward R: Reverse				

## 3. Discussion

17α-hydroxylase and 17, 20-lyase deficiencies are rare types of CAH which make up ∼1% of all CAH patients (16). "In general, the majority of patients with CAH present with hypertension and primary gonadal failure during adolescence and adulthood, however, a few individuals are reported to be normotensive at the time of diagnosis" (7, 9). The deficiency of 17a-hydroxylase is a rare reason of CAH, with little more than 100 cases reported (17). 17α-hydroxylase deficiency was first described in a 35-year-old patient in 1966 by Biglieri and co-workers, who was phenotypically female and presented with hypertension, sexual infantilism, and primary amenorrhea. In the complete deficiency of *CYP17A1*(CDC), the hormonal alterations are summed up as sex steroids and cortisol insufficiencies with mineralocorticoids excessiveness. All afflicted individuals were born with sexual infantilism and incapable of secondary sexual characteristics development (18). Isolated 17,20-lyase deficiency (ILD) was first described in boys with a disorder of sex development (DSD) (11, 19). In addition to *CYP17A1*gene, mutations in its redox partner POR gene, which encodes protein P450 oxidoreductase, can eliminate the 17,20-lyase activity (ILD) of *CYP17A1*, which is characterized by abnormal sexual development and hypertension (20). lastly, the purest form of ILD results from defects in the CYB5A gene (21), encoding the allosteric activator b5, which selectively give rise to the ILD. These individuals maintain a hint of ILD, about 10% of normal, which leads to DSD in 46, XY males.

Symptoms can be briefly listed as hypertension, primary amenorrhea, and ambiguous genitalia. In this case, unlike CDC, the levels of most 17-hydroxysteroids were elevated, suggesting exclusive impairment of ILD. Moreover, the level of cortisol as opposed to in CDC patients exists in the normal range which further rules out the impairment of 17-hydroxylase activity. The alteration in 351Leu which lies in the redox partner-binding domain of P450c17 leads to an impairment in lyase activity of P450c17 (14, 19), in other words, disrupt interactions of redox partner proteins with *CYP17A1*.

In sum, this case manifested typical feature of 17α-hydroxylase and 17,20-lyase deficiencies (e.g., hypertension and ambiguous genitalia). This study is the first paper to report an in-frame deletion which results in isolated 17, 20-lyase deficiency, and this mutation might be used for diagnosis in other patients with distinctive clinical symptoms. Identification of 17α-hydroxylase/17,20 lyase deficiency was confirmed by the particular profile of adrenal steroid levels, and further confirmation by CYP17A mutation analysis.

##  Conflict of Interest

The authors declare no conflict of interest.
